# Murine Typhus in Child, Yucatan, Mexico

**DOI:** 10.3201/eid1506.081367

**Published:** 2009-06

**Authors:** Jorge E. Zavala-Castro, Jorge E. Zavala-Velázquez, Justo Eduardo Sulú Uicab

**Affiliations:** Universidad Autónoma de Yucatán, Mérida Yucatán, Mexico

**Keywords:** Rickettsia, Rickettsia typhi, human cases, diagnosis, murine typhus, Mexico, dispatch

## Abstract

A case of murine typhus in Yucatan was diagnosed in a child with nonspecific signs and symptoms. The finding of *Rickettsia typhi* increases the number of *Rickettsia* species identified in Yucatan and shows that studies are needed to determine the prevalence and incidence of rickettsioses in Mexico**.**

Murine typhus is a worldwide febrile illness caused by *Rickettsia typhi*, which is frequently associated with exposure to reservoir animals and their ectoparasites (*1*,*2*). Patients with murine typhus often have nonspecific signs and symptoms that mimic those of common febrile illnesses. Although the disease is generally self-limited, it sometimes has complications that require hospitalization and can even cause death if the appropriate timely treatment is not administered (*3*,*4*). Antimicrobial drug treatment dramatically reduces the symptomatic period as well as the economic effects by lowering expenses and minimizing loss of productivity.

Information about murine typhus in Mexico is scarce. Recently, a study of healthy adult blood donors in Mexico City showed a prevalence of antibodies against typhus group rickettsiae (using *R. typhi* antigen) of 14% (*5*).

## The Study

In June 2007, a 3 year-old girl was brought to the emergency room of the public hospital with a fever of 3 days’ duration. Her mother reported that the child had close contact with domestic animals (cat and dog), that mice were present in the house, and that the child had flea bites on her legs and arms 2 days before the onset of fever. The illness began with a high fever (39.8°C), abdominal pain, headache, fatigue, and myalgia. The patient’s condition was diagnosed as viral pharyngitis and otitis media, and she was treated as an outpatient with nimesulide, amoxicillin, and trimethoprim; she did not respond to this treatment. Ten days after the first hospital visit, the girl was hospitalized with the diagnosis of *Salmonella typhi* infection because of serologic reactivity with O and H antigens (titers of 160 and 80, respectively). At time of admission, she had a high fever (39.8°C), arthralgia in the hands and ankles, and a petechial maculopapular rash on the thorax and extremities. She was treated with sulfonamide, cephalosporin, and acetaminophen without remission of the symptoms. Clinical laboratory evaluation showed the following: neutrophilic leukocytosis, hemoglobin 10.1 g/dL, hematocrit 30.3%, thrombocytopenia (40 × 10^6^ platelets/L [reference range 140–440 × 10^6^ platelets/L]), elevated serum alkaline phosphatase (290 U/L [reference range 35–104 U/L]), elevated lactic dehydrogenase concentration (560 U/L [reference range 100–190 U/L]), and elevated antistreptolysin O and rheumatoid factor titers.

The diagnosis of murine typhus was established by PCR for *Rickettsia* 17 kDa and citrate synthase (*gltA*) genes as described previously (*6**,**7*). Positive controls were DNA of *Rickettsia felis*, *R. rickettsii*, *R. akari*, and *R. typhi*, and 1 reaction without DNA was used as a negative control. The DNA of the controls and the patient were handled separately to avoid contamination. *R. typhi* was identified as the causal agent by restriction fragment length polymorphism analysis (RFLP) of the amplified fragment of *gltA* (382 bp) and 17-kDa gene (434 bp) by using *Alu*I as described previously (*7*,*8*) (Figure) and by comparing the DNA sequences of the *gltA* and 17-kDa gene PCR amplicons using BLAST software of the National Center for Biotechnology Information (Bethesda, MD, USA) (*9*). The sequences showed 100% identity with the corresponding *R. typhi* genes (Table).

**Figure:**
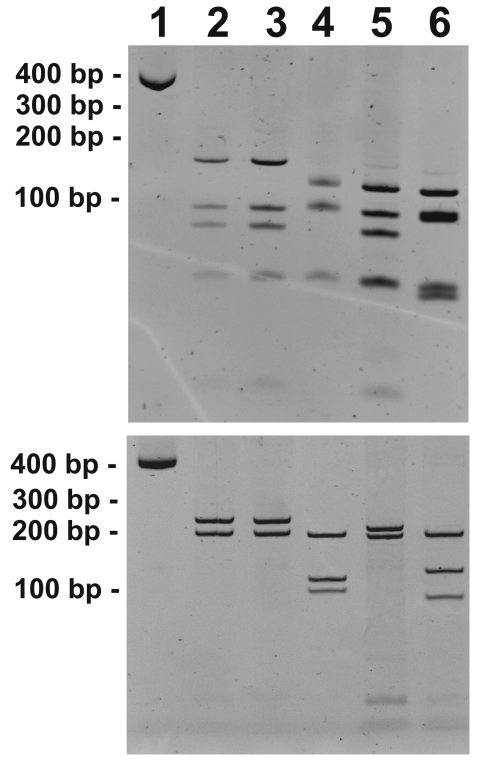
Restriction fragment length polymorphism patterns of *gltA* (A) and 17-kDa gene (B) PCR products digested with *Alu*I. Lane 1, undigested *gltA* and 17-kDa gene PCR amplicons (from blood sample of infected child); lane 2, human case; lane 3, *Rickettsia typhi*–positive control; lane 4, *R. felis*–positive control; lane 5, *R. akari*–positive control; lane 6, *R. rickettsii*–positive control.

**Table:** Methods used to diagnose human *Rickettsia typhi* infection, Yucatan, Mexico, 2007*

Sample	PCR, % identity			IFA, titer†	
	Citrate synthase (*gltA*) (382 bp)‡	17-kDa gene (434 bp)§		IgM	IgG
*Rickettsia typhi* Wilmington strain	100 (AE017197.1)	100 (M28481.1), 99 (AE017197.1)		256	128
*R. felis* URRWXCal2	NR	89 (CP000053.1)		ND	
*R. rickettsii* Sheila Smith	92 (CP000848.1)	89 (CP000848.1)		Neg	128
*R. akari* Hartford strain	91 (CP000847.1)	96 (CP000847.1)		Neg	64

*IFA, indirect immunofluorescence assay; Ig, immunoglobulin; NR, not represented in the first 100 sequences with a significant alignment using BLAST (*9*); ND, not determined; Neg, negative. †Titers refer to the reciprocal of the dilution. ‡Primers: RpCS.1258 5′-attgcaaaaa gtaccgtaaaca-3′ and RpCS.877 5′-gccccgccgtgggcaggccccc-3′. §Primers: 17-kDa 5′-gctcttgcaacttctatgtt-3′ and 5′-cattgttcgtcaggttggcg-3′.

An indirect immunofluorescence assay (IFA) was performed for serologic diagnosis; *R. akari, R. rickettsii,* and *R. typhi* antigens were fixed on slides. (A positive human serum sample control and IFA slides were provided by the Rickettsial and Ehrlichial Diseases Research Laboratory, University of Texas Medical Branch at Galveston.) As a negative control, we used a serum sample (from a healthy donor) that was negative for *Leptospira* spp. (microscopic agglutination test and PCR); rickettsiae (IFA and PCR); HIV (microparticle enzyme immunoassay and PCR); hepatitis A, B, and C viruses (microparticle enzyme immunoassay); *Toxoplasma gondii* (ELISA); and *Mycobacterium tuberculosis* (ELISA, PCR).

We examined the serum specimens for immunoglobulin (Ig) G and IgM, assessing reactivity of γ-chain–specific and μ-heavy-chain–specific secondary conjugates, respectively, with rickettsial antigens. A serum sample collected 20 days after onset of the illness was serially diluted to 1:4,096 to determine the end-point titer. IFA showed antibody reactivity with *R. typhi*, *R. rickettsii*, and *R. akari* (Table). The child was treated with intravenous chloramphenicol, 75 mg/kg per day, for 7 days; symptoms were reduced in 48 hours.

## Conclusions

In Yucatan state we have identified several cases of rickettsiosis caused by *R. felis* and *R. rickettsii*, some with fatal outcome, but not with *R. typhi* infection (*10*,*11*). Since we reported an emerging human rickettsiosis in Mexico 12 years ago, a surveillance system and educational efforts have been put in place to promote more timely diagnosis and treatment of rickettsioses. As a result of such efforts, we have also been able to detect new rickettsioses in the Yucatan Peninsula.

*R. typhi* is a bacterium that is broadly distributed around the world, and is the cause of many human infections every year (*12*,*13*). As with other febrile illnesses, children are a vulnerable population frequently exposed to this pathogen (*4*,*14*).

In Mexico, human proximity to domestic animals is common, and the habitats of both have a close relationship. In rural and suburban areas, for example, opossums, rats, and mice often inhabit backyards and houses. The potential transmission cycle of many vector-borne diseases, including rickettsioses, is evident through human exposure to ectoparasite vectors.

IFA showed serum IgM and IgG antibody reactivity with *R. typhi* and IgG antibody reactivity with *R. rickettsii* and *R. akari*. In our experience, IFA supports the diagnosis but sometimes is not conclusive because of cross-reactivity among species of the spotted fever group and the typhus group (*15*). However, we cannot exclude the possibility of other prior infections because of the living conditions and the presence of various rickettsial species in our environment in Yucatan. For that reason, we conduct PCR to diagnose human rickettsiosis. Our criteria for a confirmed diagnosis of any rickettsial infection are the same as those published by the US Centers for Disease Control and Prevention (Atlanta, GA, USA) for Rocky Mountain spotted fever: a clinically compatible case with a 4-fold change in IgG-specific antibody titer reactive with a rickettsial antigen by IFA between paired serum specimens or detection of rickettsial DNA in a blood, biopsy, or autopsy specimen through amplification of a specific target by PCR.

The identity of *R. typhi* was established not only by the RFLP pattern of the *gltA* and 17-kDa gene amplicons, but mainly by the sequence comparison with other rickettsial species. Identity was 100% for *R. typhi* (Table). Identification of the *Rickettsia* species is essential for determining the epidemiology and ecology of the transmission cycle and how the agent is maintained in nature. In addition, species identification is useful for selecting the best preventive program appropriate for each region.

This finding of an autochthonous human case of murine typhus in Yucatan Mexico and the finding of *R. typhi* in Yucatan state increases the diversity of rickettsioses identified in this ecosystem. Because rickettsioses are treatable diseases, an educational program is critical to instruct the population about these infections and their transmission cycles, as well as to inform the medical community about the rickettsial diseases that must be included in the differential diagnosis of any acute febrile illnesses in the region.
